# Rheumatoid Arthritis in the Era of COVID-19 Pandemic

**DOI:** 10.31138/mjr.31.3.257

**Published:** 2020-09-21

**Authors:** Ahmed M. Abbas, Asmaa AbouBakr

**Affiliations:** 1 Department of Obstetrics and Gynaecology, Faculty of Medicine, Assiut University, Egypt; 2 COvid-19 Research of Assiut University Association (CORAUNA) group; 3 House-Officer, Faculty of Medicine, Assiut University, Egypt

**Keywords:** COVID-19, rheumatoid arthritis, corticosteroids, chloroquine, DMARDs

In December 2019, an outbreak of a new coronavirus infection by SARS-COV-2 in China had quickly become a global health emergency, causing coronavirus disease-19 (COVID-19).^1^ The current treatment of COVID-19 is mainly supportive, and no specific antiviral therapy is available.^2^ Patients under immunosuppressive treatment are easy prey for COVID-19.^3^ The Coronaviridae family is a single-stranded RNA genome. Viral RNA released in host cell cytoplasm starts processes of translation, transcription and replication, leading to downregulation of the Angiotensin-converting enzyme 2 (ACE2) receptors, as the immune system responds by expression of ACE2 of the host cell surface that binds to spike glycoprotein of the viral envelope which in turn result in the release of angiotensin 2. This will stimulate type1a angiotensin 2 receptors in the lung, increasing pulmonary vascular permeability and lung damage.^4^

Patients with rheumatoid arthritis (RA) are fragile and more vulnerable to infection compared to the general population owing to their impaired immunity and iatrogenic effect of immunosuppressive drugs.^5^

Corticosteroids are used in the treatment of RA due to their anti-inflammatory effects. However, they have multiple adverse effects and a significant risk of infectious diseases. There is no clear evidence of the beneficial effect of steroids on COVID-19.^6^ A recently published systematic review included five studies for evaluating the role of corticosteroids in COVID-19; three of them have shown benefit, while two studies shown no benefit and there was a suggestion of significant harm in critical cases in one sub-study.^7^ Therefore, the role of corticosteroids is still controversial. Current interim guidance from the World Health Organization (WHO) in acute respiratory distress is not to give corticosteroids unless indicated for other reasons.

Theoretically, NSAIDS worsens the clinical picture of patients with COVID-19 due to overexpression of ACE2 receptors.^5^ However, Capuano et al. concluded that there is no evidence suggesting a correlation between NSAIDs and a worsening of infections including COVID-19.^8^

Using conventional synthetic Disease-modifying anti-rheumatic drugs (csDMARDS) without corticosteroids decreases the risk of mild infection based on a longitudinal study of a population-based RA cohort.^9^ Rheumatoid patients using biological (bDMARDS) are associated with an increased risk of infection compared to csDMARDS. In contrast, many drugs used to treat RA are possible therapy of COVID-19 as chloroquine, hydroxychloroquine, IL-6 inhibitors, baricitinib and TNF-inhibitors.^10^

Although there are many potential drugs suitable for reducing inflammation in COVID-19, the anti-TNF antibodies tocilizumab, infliximab or adalimumab are potentially effective, widely available, and have a good safety profile.^11^ In an observational trial in RA patients with serious sepsis, the risk of infection and death was reduced in patients on TNF inhibitors compared with those on csDMARDS.^12^

Chloroquine and hydroxychloroquine, besides their immunomodulation effect on many rheumatologic diseases, have antiviral properties.^13^ Their mechanism of actions is through increasing PH for viral invasions, inhibition of the toll-like receptors, and interference with terminal glycosylation of ACE2 receptors. Various combination protocols with antivirals are used in China against SARS-COV-2 infection.^14^ Viral spike protein induces a TNF-α-converting enzyme-dependent shedding of ACE2 ectodomain, so TNF-inhibitors are prescribed in SARS-COV infection.^5^ There is an increasing number of studies that show no benefit of chloroquine in patients with COVID-19.^15^ On the other hand, there is no controversy whether patients with RA should continue their treatment with Chloroquine and hydroxychloroquine regardless of the risk of COVID in accordance with ACR recent recommendations.^16^

Conclusively, the COVID-19 pandemic is an emergency that threatening the general population, especially those with disturbing immune diseases such as RA. Treating rheumatoid patients with corticosteroids worsen the SARS-COV-2 infection, but they can be used as bridging therapy. Use of conventional synthetic, targeted synthetic, and biological DMARDS also have a great risk of infection; however, they are effective and used to control RA. Chloroquine and hydroxychloroquine are considered in the management protocol of COVID-19 and RA. However, they have increasingly controversial data regarding their effect in COVID-19 patients.^17^
